# Imaging findings of pulmonary manifestations of chronic granulomatous disease in a large single center from Shanghai, China (1999–2018)

**DOI:** 10.1038/s41598-020-76408-4

**Published:** 2020-11-09

**Authors:** Qiong Yao, Qin-hua Zhou, Quan-li Shen, Zhong-wei Qiao, Xiao-chuan Wang, Xi-hong Hu

**Affiliations:** 1 Department of Radiology, Children’s Hospital of Fudan University, Shanghai, 201102 China; 2 Department of Allergy and Clinical Immunology, Children’s Hospital of Fudan University, Shanghai, 201102 China

**Keywords:** Diseases, Medical research

## Abstract

Chronic granulomatous disease (CGD) is characterized by recurrent infections and granuloma formation in multiple organs, especially the lung. We aimed to investigate pulmonary manifestations by computed tomography (CT). In total, 100 patients with 117 episodes of pulmonary infection were included. Chest CT scans of every episode were analyzed. Random nodules were the most common findings (79.49%), followed by ground-grass opacities (74.36%), focal consolidations (62.39%), and masses (59.83%). Cavities (12.82%) and multiple small abscesses (17.09%) could be found in the consolidations and masses. CT revealed interstitial pneumonia with tree-in-bud opacities (17.09%), interlobular septal thickening (23.08%) and emphysema (35.04%), which were more severe in the bilateral upper lobes. Mediastinal and hilar lymphadenopathy (78.63%) and axillary lymphadenopathy (65.81%) were common. Fungal infection (n = 27) was the most common and presented with multiple nodules and masses. Approximately 1/4 of fungal infections had interstitial pneumonia. In *Staphylococcus aureus* (n = 6) and *Klebsiella pneumoniae* (n = 3) infections, large areas of consolidation were common. In tuberculosis infection, the pulmonary infections were more severe and complex. For *Bacillus Calmette-Guérin* disease, left-sided axillary lymphadenopathy was a characteristic manifestation. CT images of CGD demonstrated variable pulmonary abnormalities. The main infectious organisms have unique imaging features.

## Introduction

Chronic granulomatous disease (CGD) is a rare primary immunodeficiency caused by mutations in the nicotinamide adenine dinucleotide phosphate (NADPH) oxidase gene. It causes lack of oxygen free radicals needed to kill microorganisms. The disease is estimated to occur as 1 in approximately 200,000–250,000 live births in the USA^1,2^. The characteristic manifestation of CGD is recurrent infection and an inflammatory response with granuloma formation. The most common organisms causing respiratory infection are *Aspergillus*, *Staphylococcus aureus* (*S. aureus)*, *Klebsiella pneumoniae*, *Streptococcus pneumoniae*, *Burkholderia cepaciam*, *Nocardia species* and *Candida albicans*^1^. In China, Bacillus Calmette-Guérin (BCG) is injected into every newborn, and the incidence of BCG infections in this group of patients is also high.

The lung is the most common site of involvement (95.9%), followed by lymph nodes (58.5%), skin and soft tissue (45.4%), the intestinal tract (43.1%), and the perianal region (38.5%)^3^. Various manifestations of pulmonary lesions can be found in CGD patients. The most frequent manifestations are due to infection, such as pneumonia, which could progress to abscesses or interstitial inflammation^4,5^. Inflammatory and autoimmune responses include granuloma formation and pulmonary fibrosis, which usually can be found in longer courses of the disease. In addition, pleural thickening and chest wall invasion are commonly caused by adjacent pulmonary infections^6,7^. As early diagnosis and aggressive management (antibiotic agents, interferon γ and hematopoietic stem cell transplantation) have been widely achieved, honeycomb lung and respiratory failure are rare in children today.

Since the most common site of involvement in CGD is the lung, it is important for clinical doctors to recognize various performances on chest CT images, especially with different pathogens. However, due to its low morbidity, there are few studies regarding the radiologic manifestations of CGD in a large sample. The aim of this study was to analyze the radiographic findings of the lung of CGD patients in the last 20 years and summarize the imaging characteristics.

## Results

### Demographic and clinical features

The demographic and clinical features of the subjects involved in the study are summarized in Table 1. One hundred CGD patients with 117 episodes of infection were included in the study. Ninety-six (96%) patients were men, and 4 (4%) were female (32.25 ± 42.14 months, IQR 3–33 months). A total of 117 cases of infection in the 100 patients were identified (100 associated with *CYBB*, 10 with *CYBA*, 3 with *NCF1* and 1 with *NCF2* mutations), and all had experienced at least 1 infectious episode (IQR 1–3). The average stimulative index was 5.37 ± 5.33 (4.56 ± 4.30 for *CYBB*, 8.56 ± 5.04 for *CYBA*, and 15.15 ± 13.93 for *NCF1*).

**Table 1:** Demographic information and clinical data.

**Sex**	
Male	96
Female	4
**Gene type**	
CYBB	87
CYBA	7
NCF1	2
NCF2	1
Unknow	3
Age (month, IQR)	32.25 ± 42.14 (3–33)
Periods of infection	117
**Main infectious pathogens**	
Staphylococcus aureus	6
Klebsiella pneumoniae	3
G (+)	10
Gm (+)	17
TB	7
BCG	69

All the microbial laboratory exams were reviewed. Totally 27 patients were identified as invasive fungal infection (IFD) according to the serum G ((1–3)-β-d-glucan) tests and GM (galactomannan) test. 10 (37.04%) were G positive and 17 (62.96%) were GM positive. Cultural evidence for fungi infection was rare in this study. Only 3 strains of fungi (1 with *Aspergillus* and 2 with *Candida albicans*) were isolated from sputum and identified as confirmed IFD, and the rest 24 cases were probable IFD. Twenty strains of bacteria were isolated from sputum, including 6 strains of *S. aureus*, 3 strains of *Klebsiella pneumoniae*, 3 strains of *Streptococcus pneumoniae*, 2 strains of *Escherichia coli*, 2 strains of *Moraxella catarrhalis*, 1 strain of *Salmonella typhimurium*, 1 strain of *Enterobacter cloacae*, 1 strain of *Haemophilus influenzae*, and 1 strain of *Burkholderia cepacia*. Seven patients were positive in the T-Spot and PPD tests, suggesting tuberculosis (TB) infection. BCG disease was diagnosed in 69 patients, among whom 15 were PPD positive. Among the 21 patients with lymph node puncture, 6 presented with necrosis, lymph cell infiltration, epithelioid granuloma formation, and positive acid-fast smear and were subsequently diagnosed with BCG disease.

Some patients were more likely to be infected with two or more pathogens. In the 24 probable IFD patients, four were accompanied by *S. aureus* (n = 2), TB (n = 1), and *Klebsiella pneumoniae* (n = 1).

### CT manifestations in the whole population

**Figure 1:**
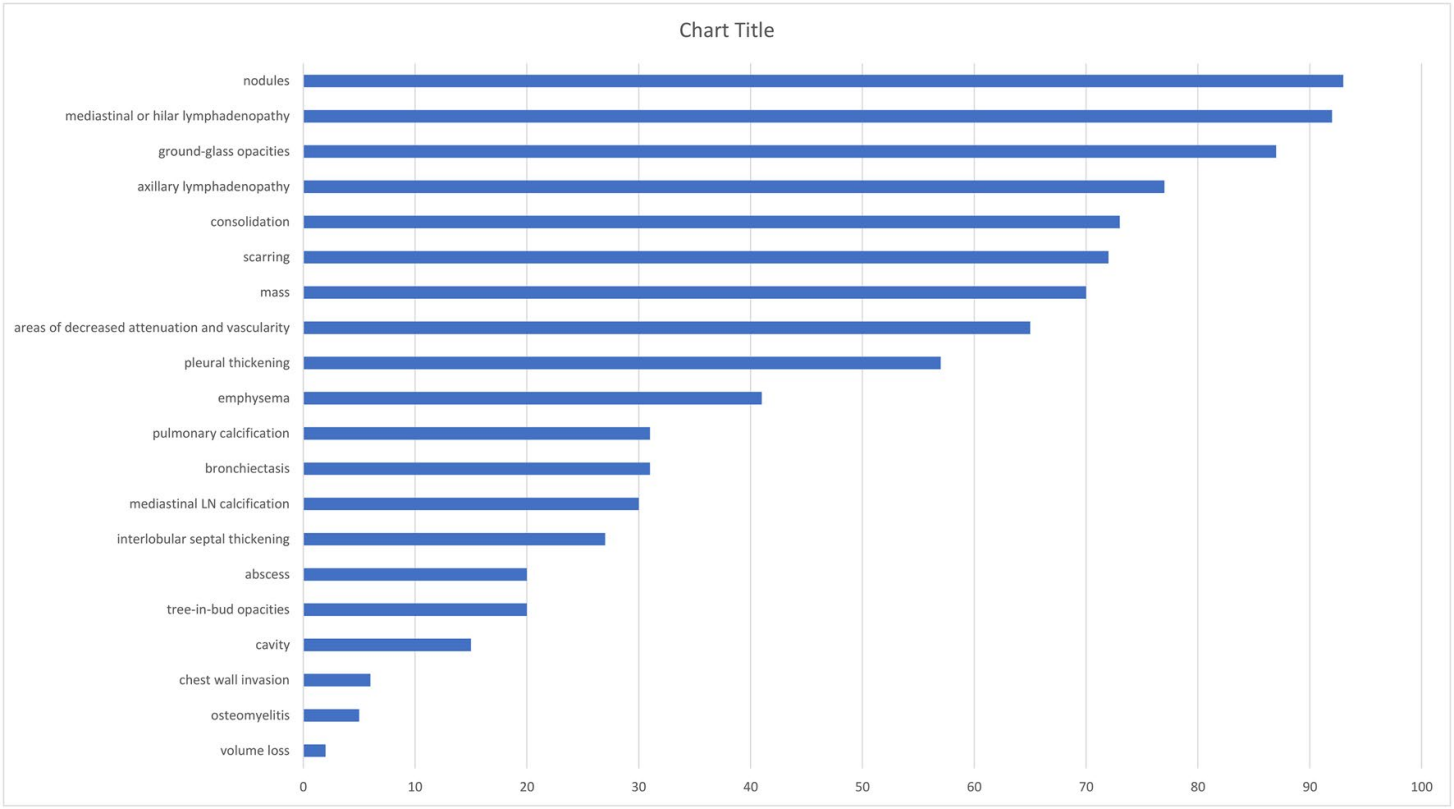
Spectrum of pulmonary lesions in CGD Patient in order of frequency.

The main CT findings of the pulmonary lesions in the acute stage of infection are listed in Fig. 1 and arranged based on the number of affected patients. Among the 117 episodes of infection, random nodules were the most common findings, which were detected in 93 (79.49%) episodes, ranging from 1 to 3 cm in diameter and mainly in the bilateral lower lobes. Other main abnormalities in the pulmonary parenchyma included ground-grass opacities (87/117, 74.36%), focal consolidations (73/117, 62.39%), and masses (70/117, 59.83%). On CT, ground-glass opacities predominately presented in the lower lobes and were always associated with consolidations. Cavities (15/117, 12.82%) and multiple small abscesses (20/117, 17.09%) were found in the focal consolidations and masses. The CT scans revealed interstitial pneumonia with tree-in-bud opacities (20/117, 17.09%), interlobular septal thickening (27/117, 23.08%) and paraseptal emphysema (41/117, 35.04%), which were extensive and more severe in the bilateral upper lobes. Pulmonary scarring (72/117, 61.65%) was mild and localized in the lower lobes in most patients with slight bronchiectasis (31/117, 26.50%). Older patients with a longer course of disease showed an increased level of pulmonary fibrosis. Only 2 patients presented with extensive pulmonary fibrosis and volume loss of the related pulmonary lobe in the chronic infection phase, accompanied by pulmonary artery hypertension confirmed by echocardiography. Mediastinal and hilar lymphadenopathy (92/117, 78.63%) and axillary lymphadenopathy (77/117, 65.81%) were common, with calcification identified in almost 1/3 of the patients. Pleural thickening (57/117, 48.72%) was common, but none showed a large amount of effusion. Chest wall invasion was found in 6 (5.13%) episodes, presenting as osteomyelitis of the ribs and soft-tissue abscesses of the chest wall.

### CT findings based on different pathogens

In patients with different kinds of evidences of pathogen infection, the CT findings were analyzed separately to study them, which are summarized in Table 2.

**Table 2:** Number and percentage of pulmonary lesions in CGD patient with different pathogens.

	S (%) n = 6	K (%) n = 3	Fungi (%) n = 27	TB (%) n = 7
Consolidation	2 (33.33)	3 (100.00)	17 (62.96)	5 (71.43)
Ground-glass opacity	4 (66.67)	3 (100.00)	22 (81.48)	4 (57.14)
Nodules	4 (66.67)	2 (66.67)	21 (77.78)	5 (71.43)
Mass	2 (33.33)	2 (66.67)	20 (74.07)	4 (57.14)
Cavity	2 (33.33)	1 (33.33)	3 (11.11)	1 (14.29)
Tree-in-bud opacities	0	0	3 (11.11)	0
Bronchiectasis	1 (16.67)	0	7 (25.93)	3 (42.86)
Emphysema	1 (16.67 )	0	7 (25.93)	4 (57.14)
Scarring	3 (50.00)	2 (66.67)	13 (48.15)	5 (71.43)
Mediastinal or hilar lymphadenopathy	5 (83.33)	2 (66.67)	24 (88.89)	6 (85.71)
Pulmonary calcification	1 (16.67)	1 (33.33)	7 (25.93)	3 (42.85)
Mediastinal lymph node calcification	0	0	5 (18.52)	3 (42.85)
Pleural thickening	2 (33.33)	2 (66.67)	11 (40.74)	4 (57.14)

S, *Staphylococcus aureus*; K, *Klebsiella pneumoniae*; TB, tuberculosis.

**Figure 2:**
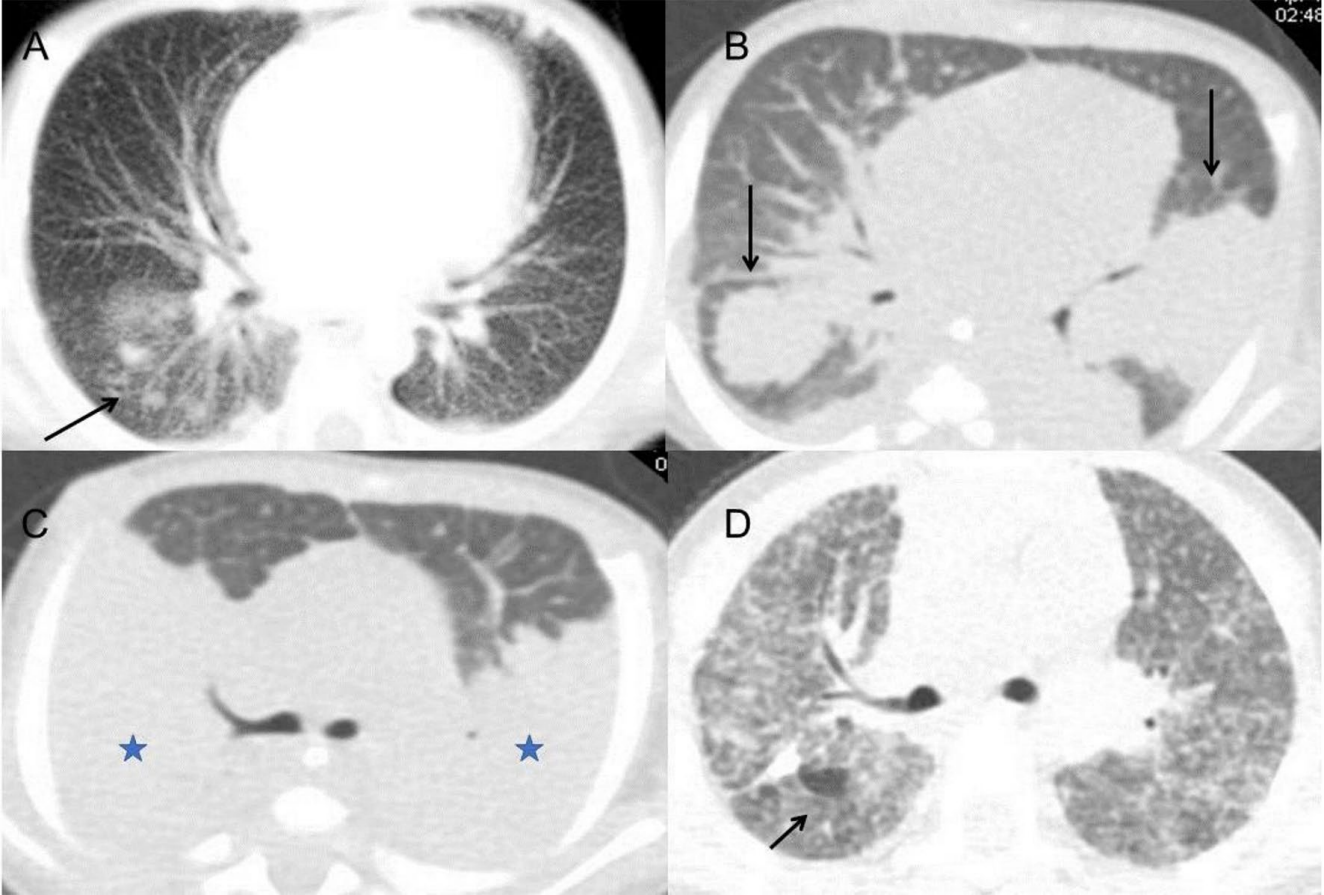
CT images of fungal infection. (**A**,**B**) Multiple nodules (**A**, long arrow) and masses (**B**, long arrow) were found in the bilateral lower lobes on axial CT images. (**C**) Chest CT showed bilateral large areas of consolidation (star) in a 1-year-old boy. (**D**) In a 2-year-old boy, extensive interstitial pneumonia with tree-in-bud opacities, interlobular septal thickening and paraseptal emphysema (short arrow) were demonstrated.

**Figure 3:**
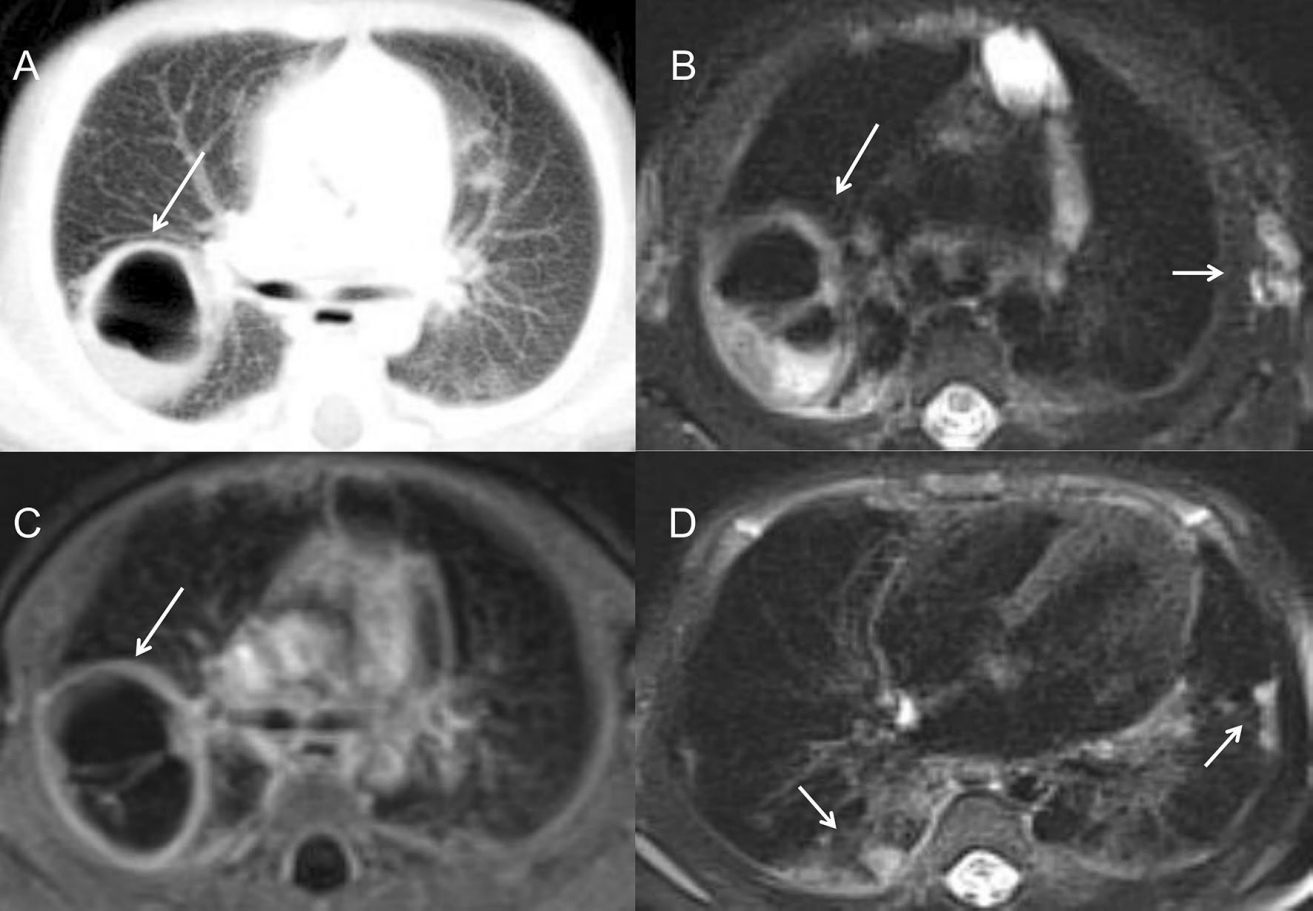
A 1-month-old boy with aspergillus infection. (**A**) On chest CT, a large cavity with thin and smooth wall was found in the right upper lobe, ranging 3 cm in diameter (long arrow). (**B**) On T2WI image, the linear separations and gas liquid leveling were found inside (long arrow). Multiple enlarged lymph nodes were found in the left axillary area (short arrow). (**C**) On post-contrast T1WI image, the wall of the cavity was obviously enhanced (long arrow). (**D**) Multiple pulmonary nodules were also seen in the bilateral lower lobe beneath the pleura (short arrow).

Fungal infection was the most common in CGD patients. Multiple nodules and masses in a random distribution were the most features in fungal infection (Fig. 2A,B). In the whole IFD group (n = 27), the incidence of nodules was 77.78%; they were regular in shape, multiple in the bilateral lung fields, and some with a halo sign. Large areas of consolidation were usually found in the lower lung fields (Fig. 2C). Large cavities with thin and smooth walls were only found in 3 episodes, ranging from 1 to 3 cm (Fig. 3). We did not find the air crescent sign in our cohort. Fungal infections were usually identified as the first pathogen and presented with multiple large masses in the bilateral lung fields in the infantile period. Mediastinal or hilar lymph node enlargement also had a relatively high incidence, usually in mild size (10–15 mm) and with a low incidence of calcification. Almost 1/4 of fungal infections presented with extensive interstitial pneumonia with a longer course of disease, diffuse tree-in-bud signs and ground-glass opacities (Fig. 2D). Pulmonary scarring was mild and localized.

Among the 10 G positive and 17 GM positive patients, only 3 strains of fungi were isolated from sputum, 1 with Aspergillus, and 2 with Candida albicans. We future separated the 27 IFD patients into 2 groups: confirmed IFD group and probable IFD group. We compared the radiological findings of 2 groups and found no obvious difference.

**Figure 4:**
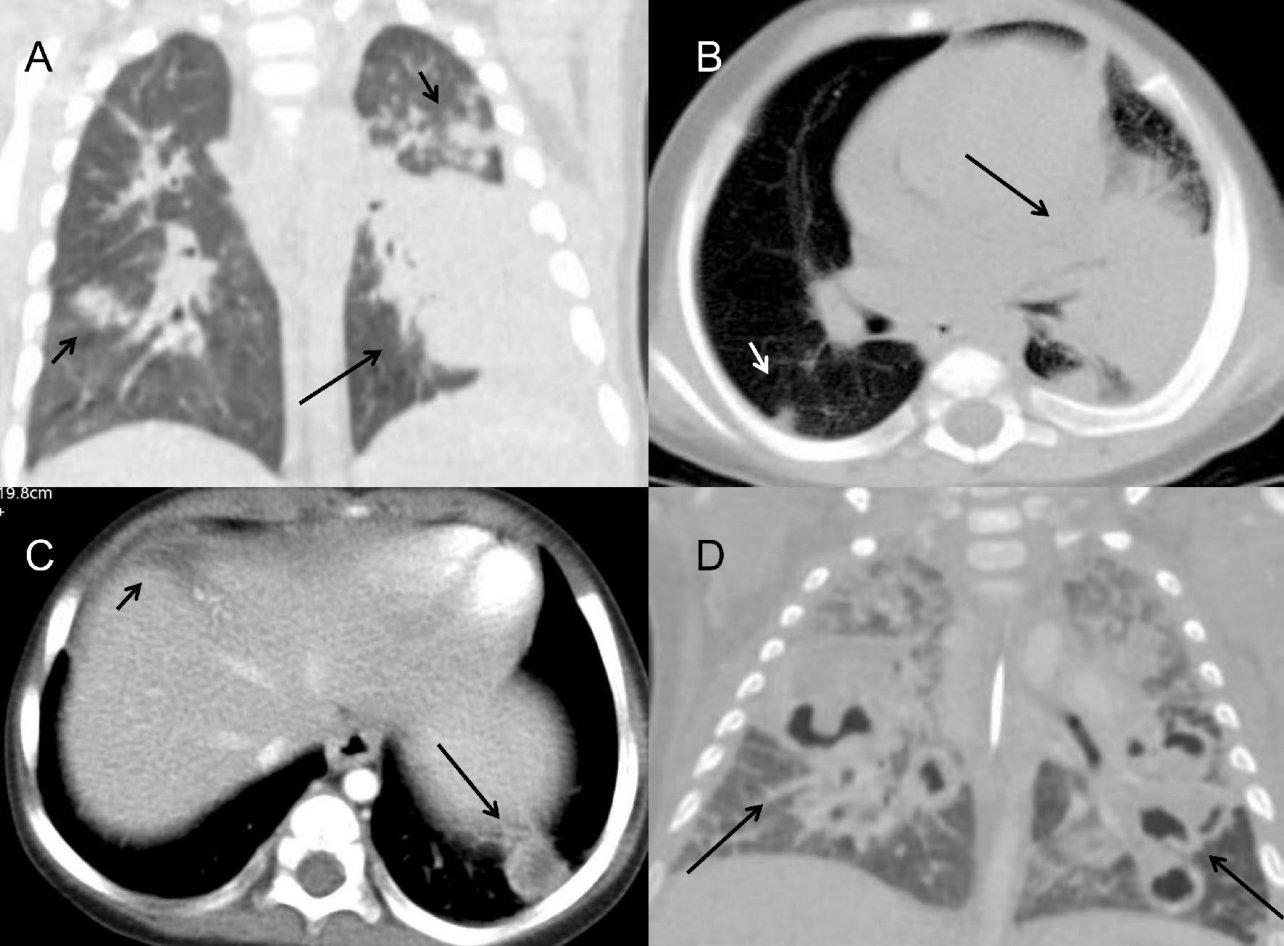
Bacterial infection in CGD patients. (**A**,**B**) A 5-month-old boy with klebsiella pneumoniae infection showed a large area of consolidation in the left lower lobe on axial and coronal reformat CT images (long arrow). Multiple nodular lesions in the bilateral lung fields were found (short arrow). (**C**) A 1-year-old boy with Staphylococcus aureus infection. Chest CT showed the small nodular lesion with abscess inside in left lower lobe (long arrow). The low-attenuated lesion in the left lobe of liver was seen, suggesting the possibility of hepatic abscess (short arrow). (**D**) A 1-year-old boy with Staphylococcus aureus infection. On CT, masses and patchy consolidations were found in the bilateral lung fields with multiple small cavities formation (long arrow).

In *Klebsiella pneumoniae* infections (n = 3), all showed consolidation of the whole unilateral upper lobe (Fig. 4A,B). In *S. aureus* infections (n = 6), large areas of consolidation were the most common lesions, mainly in the bilateral lower lobes, with abscesses and thickened wall cavities inside (Fig. 4C,D). All patients had mild mediastinal or hilar lymph node enlargement (10–20 mm) without calcification. Slight pulmonary fibrosis was found.

**Figure 5:**
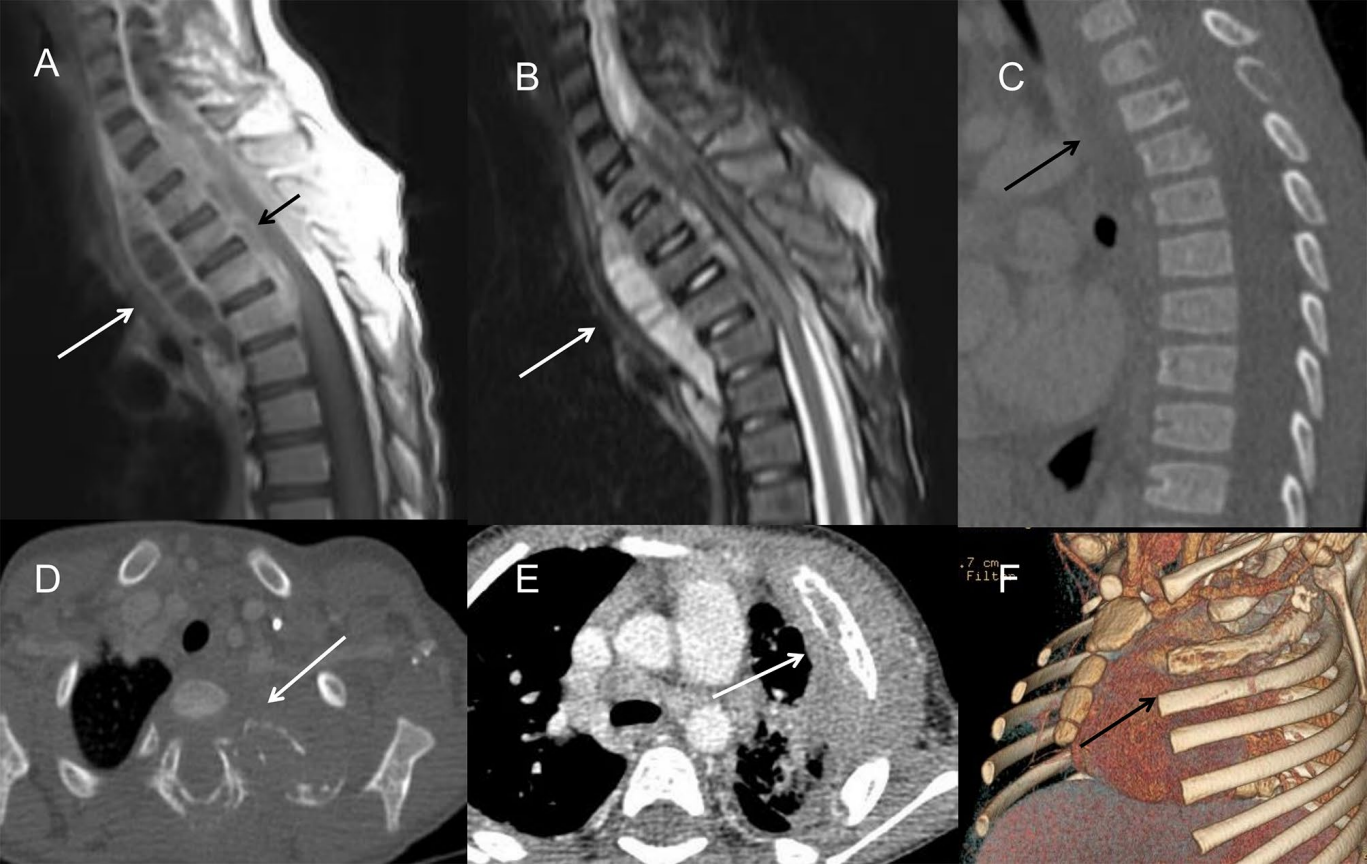
(**A**–**D**) A 3-year-old boy with infection of TB. (**A**) On CT, the 3rd thoracic vertebra was slightly compressed and the 3rd and 4th thoracic vertebra had multiple small areas of osteolysis (long arrow). (**B**) On T2WI image, paravertebral abscess extending into the spinal canal and compressing the thoracic spinal cord could be found (long arrow). (**C**) The abscess wall was markedly enhanced on post-contrast T1WI sequence (long arrow). (**D**) On axial CT image, the osteolysis and expansion of the left first rib and vertebral plate were found (long arrow). (**E**,**F**) A 2-year-old boy with fungi infection. On CT images, osteomyelitis of the left 2nd rib and surrounding soft tissue swelling were showed (long arrow). The adjacent lung infection and pleural thickening were also noticed.

In TB infection (n = 7), the pulmonary infections were more severe and complicated. Masses and nodules of different sizes in the bilateral pulmonary field were identified in 6 patients, while nodular calcification was observed in 3 patients. Some masses and nodules had various levels of heterogeneous enhancement, indicating the existence of caseous necrosis. Large areas of consolidation and ground-grass opacity were common in the bilateral fields, some with multiple small cavities and necrosis within. Two patients presented extensive pulmonary fibrosis and severe emphysema, extensive bronchiectasis, and ipsilateral pulmonary volume loss. Mediastinal and hilar lymphadenopathy enlargement were remarkable in 2 patients, 5 cm in diameter, leading to adjacent bronchostenosis and pulmonary atelectasis. In a 3-year-old boy with TB, paravertebral abscesses extending into the spinal canal and osteomyelitis of the 3rd and 4th thoracic vertebra could be found (Fig. 5A–D). BCG infection was characterized by ipsilateral axillary lymph node enlargement after vaccination and calcifications, which are usually present at 6 months of age, some with surrounding rim enhancement. No obvious pulmonary infection was identified in this cohort.

In summary, random nodules were identified in 32 patients, among whom 21 (65.66%) had a fungal infection, followed by TB (5/32, 15.63%), *S. aureus* (4/32, 12.5%), and *Klebsiella pneumoniae* (2/32, 6.25%) infections. GGOs presented mostly in 33 patients, 22 (42.85%) with a fungal infection, 4 (12.12%) with *S. aureus*, 4 (12.12%) with TB, and 1 (9.09%) with *Klebsiella pneumoniae*. Cavities were present in 7 patients, 3 (42.85%) with fungal infections, 2 (28.57%) with *S. aureus* infections, 1 (14.29%) with a *Klebsiella pneumoniae* infection, and 1 (14.29%) with a TB infection. Pulmonary fibrosis was found mainly in 23 patients, 13 (56.52%) with a fungal infection, 5 (21.74%) with TB, 3 (13.04%) with *S. aureus*, and 2 (8.70%) with *Klebsiella pneumoniae*. All 3 patients with interstitial pneumonia presented with a fungal infection.

### Interobserver correlations

Agreement between the two radiologists was strong in determining the main chest CT features. For the diagnosis of pathogens, the agreements for fungal infections (κ value of 0.89) and BCG infection (κ value of 0.76) were good. There was moderate agreement for TB infections (κ value of 0.52) and weak agreement in the diagnosis of bacterial infections (κ value of 0.38).

## Discussion

CGD is considered a hereditary immunodeficiency disease characterized by defective granulocyte respiratory bursts due to impaired NADPH activity, resulting in recurrent infections in multiple systems. Today, at least six different genetic defects have been identified in CGD^8^. Among them, X-linked recessive defects in gp91^phox^ (*CYBB*) are the most common form (> 80%). Other less frequent mutations caused by autosomal recessive defects include p22phox (*CYBA*), p47phox (*NCF1*), p67phox (*NCF2*), and p40phox (*NCF4*). The severity of infection can be related to the gene type and the level of the stimulative index. *CYBB* mutations usually present with earlier and worse clinical manifestations than the other types^9^. In our research, the average stimulative index of *CYBB* was found to be much lower than that of *CYBA* and *NCF1,* indicating more severe granulocytic disability.

The lungs have been found to be the most affected organs, with various and complex manifestations in CGD patients^1,10,11^. Radiologic findings can reveal multiple manifestations for the evaluation of disease progress. The findings of this paper were consistent with previous studies, including infiltration, consolidation and pulmonary nodules in acute infection^12^. Nodules were most common in CGD, ranging from 1 to 5 mm with or without a halo sign, which were usually associated with fungal infection and are distinct from lymphoma or metastatic tumors. The lower lobes of both lungs are the most frequent location of consolidation and ground-grass opacity. Abscesses and cavity formation have been reported to be found in up to 20% of patients^13^. Cavities usually present with a smooth wall with no mural nodule. Lung infections in CGD patients typically have a protracted course with a tendency to recurrence, which may be complicated with pulmonary fibrosis and bronchiectasia^14,15^. Fibrotic changes and bronchiectasis are usually seen in patients above 8 years old, mostly in the lower lobes, probably caused by chronic infection and inflammatory response^5^. In our cohort, almost 2/3 of patients only had a mild degree of pulmonary scarring and local bronchiectasis, possibly because the children involved in the study were at a younger age and were under effective clinical treatment. Persistent hilar or mediastinal lymphadenopathy was detected in most of our patients, especially left axillary lymphadenopathy, which is highly suggestive of BCG infection. Chest wall invasion, including osteomyelitis of the ribs and vertebral bodies, has also been reported as a specific manifestation associated with adjacent pulmonary infection^4,13,16^. *Aspergillus* is the predominant source of chest wall osteomyelitis (Fig. 5E,F), followed by TB^17^. Six of our patients had chest wall invasion, with lung masses and consolidation in the adjacent lung lobes. Chest CT may reveal osseous erosion with periosteal reaction and soft tissue masses. MRI can display bone marrow signal abnormalities and surrounding soft-tissue inflammation, which can serve as a useful tool to make an accurate assessment of the severity of the disease^18^.

For patients with CGD, each infection is potentially life-threatening, yet it has always been a challenge to identify the pathogen responsible for the infection. Lung biopsy and bronchoscopy might be chosen when the empiric therapy is useless. Radiologic findings are of great importance in providing differential diagnostic clues and improving survival rates. Meanwhile, some radiologic findings target only certain species of infection. Fungal infections, mainly caused by *Aspergillus*, account for an important proportion of CGD at any age^19^. Winkelstein reported that *Aspergillus* was a leading cause of death (1/3)^1^. Cohen reviewed 245 patients, 20% of whom had fungal infections, the majority of which (78%) were caused by *Aspergillus*^20^. The positive ratio of fungal culture is not high. Indirect evidences, including G and GM tests, characteristic chest CT features, and patients’ responses to antifungi therapy, can suggest the infection of fungi in the clinical work^3^. In our study, we identified three features of fungal infection: (1) the incidence of nodules was higher in fungal infection than in bacterial and TB infection. Multiple small nodules with regular shapes and halo signs are highly suggestive of fungal infection. Meanwhile, the effectiveness of anti-fungi treatment in our cohort also supported the diagnosis. Some researchers have presented strong evidence arguing that small nodular lesions in CGD are highly suggestive of pulmonary fungal infection, usually taking place in the early stage^21^. (2) Fungal infections were also found to be responsible for the initial onset of pulmonary infection in the infantile period, which presented with multiple large masses in the bilateral lung fields and could be misdiagnosed as lung abscesses or tumor metastasis. (3) Some patients with fungal infection presented with long-term diffuse interstitial lung diseases, mimicking hypersensitivity pneumonitis, which is rarely found in infections from other types of pathogens^22,23^.

As indirect evidences for fungi infection, both G and GM test have moderate sensitivity and high specificity. The combination of both methods can further improve the diagnostic efficacy and make the treatment more precise. In a study of patients with IFD, the negative predictive value of G test to diagnose *Candida albicans* was 100%, and that of GM test to diagnose *Aspergillus* was 91.2%^24^. In a review of 493 patients with high risk of IFD, the combination of G and GM tests could raise the sensitivity to 69% and specificity to 98%^25^. In our study, there was no obvious difference in the chest CT features between the confirmed and probable IFD group, which could prove the accuracy of G and GM test.

Among bacterial infections, *S. aureus* and *Klebsiella pneumoniae* have been found with a higher incidence in the literature as well as in our cohort^26^. The most common findings included large areas of consolidation and infiltration, usually involving a whole lung segment or lobe. The areas of consolidation generally presented as heterogeneous on enhanced CT, sometimes with multiple ring-enhancement, indicating necrosis and abscess formation. Mediastinal or hilar lymph node enlargement was not remarkable and usually without calcification.

CGD patients have been shown to be vulnerable to the mycobacteria of the TB complex, such as BCG or more severe pathogenic TB, but not to other mycobacteria, such as the *M. avium* complex and *M. kansasii*^19,27^. CGD patients from Asia, Africa, and Latin America tend to have a higher TB incidence, probably reflecting elevated exposure to the pathogen. For TB infection, early-stage infection was rarely found, and the CT findings usually included various manifestations in the later stages of disease, including caseous necrosis in the masses and nodules. Hilar or mediastinal lymph node enlargement was much more severe than other types of pathogens, leading to adjacent bronchostenosis and pulmonary atelectasis. These features are difficult to find in other pathogens, such as fungal and bacterial infections. Severe pulmonary fibrosis is another imaging feature of TB. Some patients presented with ipsilateral pulmonary volume loss, which is not common in other infections. We think for patients with a prolonged course, caseous necrosis in the masses and nodules, severe fibrosis and remarkably enlarged mediastinal lymph nodes, TB should be considered. Miliary TB due to hematogenous spread has rarely been observed^27^. In China, all neonates are inoculated with the BCG vaccine, where ipsilateral axillary lymph node enlargement and calcification is a common sign of infection, which can spread to mediastinal and/or hilar lymphadenopathy along the lymphatic drainage system. The incidence of BCG is much higher than that of TB.

The results showed a high level of agreement between the two radiologists in the diagnosis of typical CT manifestations. The fungal and mycobacterial infections had unique CT features, so the agreement between the two radiologists was also good. The discrepancy in the discrimination between bacterial infections may be attributed to the lack of specific radiographic findings.

Lifelong treatment and regular follow-up could help control the infection in CGD patients^19^. In most cases, appropriate antibiotics and antifungal treatment have proven effective against infections, with a marked increase in the survival rate up to 90% today. Approximately 1/4 of our patients received hematopoietic stem cell transplantation, which significantly alleviated the primary infectious and inflammatory lesions. At present, hematopoietic stem cell transplantation has proven to be the only treatment to cure CGD and reverse organ dysfunction^28^.

The main limitation of our research was the lack of microbiology evidences of pathogens, including the low positive rate of bacterial and fungal culture, as well as the BCG strains. As an invasive operation, the ratio of lung biopsy was quite low. Most of the fungi, TB, and BCG infections were diagnosed by the combination of laboratory test, clinical presentation and radiological features. Therefore, we propose to start new methods of test, like metagenomics sequencing, on CGD patients to better discover the possible pathogens.

## Patients and methods

### Demographics

This study was approved by the ethics committee of the Children’s Hospital of Fudan University. Informed consent was obtained from all participants' parents. All methods were carried out in accordance with relevant guidelines and regulations. We retrospectively reviewed 150 patients with CGD diagnosed in infancy or childhood, and at last 100 patients with pulmonary infection were included. The patients were treated in our institution from 1999 to 2018. In total, 117 episodes of pulmonary infection were identified, and CT scans with the most severe manifestations on the acute stage in every episode were included in the assessment.

### Laboratory test

The diagnosis of CGD was made according to defective respiratory bursts detected by the dihydrorhodamine-1,2,3 (DHR) test, decreased protein level of gp91 (flow cytometry-based extracellular staining with Moab 7D5), and mutations detected by Sanger sequencing of target genes (*CYBB, CYBA, NCF1, NCF2* and *NCF4*)^3^. The results of G and GM tests in serum were used as microbial evidence for probable IFD^3^. The GM index was defined as positive when > 0.5. The G index was defined as positive when > 100 pg/ml^29^. The GM test is highly sensitive to Aspergillus infection. Positive culture of fungi is used for diagnosis of confirmed IFD. The PPD skin test and T-spot test were used for the diagnosis of TB. A definitive diagnosis of BCG disease was made by staining for acid-fast bacilli, while a probable diagnosis was made according to the history of BCG vaccination without microbiological evidence. The PPD test is highly positive in disseminated BCG disease^30^. Lymph node puncture was performed on 21 patients.

### CT examination and parameters

All CT scans were obtained by a 64-detector scanner (GE Healthcare, Princeton, NJ). The images were acquired at end-inspiration from the apex of the lung to the diaphragm at 80–100 kV and 80–120 mA. All CT scans had a reconstruction slice thickness of 0.625 mm. Two experienced chest radiologists with more than 5 years of experience in pediatric radiological research recorded the CT findings. If there were discrepant readings, they discussed and obtained a decision by consensus. The observers evaluated consolidations, nodules, ground-glass opacities, masses, abscesses, cavities, tree-in bud opacities, interlobular septal thickening, pulmonary scarring, bronchiectasis, emphysema, pleural thickening, mediastinal or hilar lymphadenopathy, axillary lymphadenopathy, chest wall invasion, and calcification in the mediastinal or hilar lymph nodes and pulmonary parenchyma. The radiographic findings were defined according to the Fleischner Society nomenclature^31^. Consolidation appeared as a homogeneous, large lesion of the lung. A nodule was defined as a rounded opacity, well or poorly defined, up to 3 cm in diameter, while a mass was defined as more than 3 cm in diameter. Ground-glass opacity was defined as a hazy lesion of increased opacity in the pulmonary parenchyma, with bronchial and vascular margins visible within. Bronchiectasis was defined as localized or diffuse dilatation of a bronchus. Cavities presented as gas-filled spaces within pulmonary consolidation, a mass, or a nodule. Emphysema was defined as a focal area of low attenuation, usually without visible walls. Mediastinal or hilar lymphadenopathy was defined by a short-axis lymph node diameter of more than 10 mm.

### Statistical analysis

Data were presented in a descriptive way. Continuous parameters are presented as the means ± standard deviations and interquartile ranges (IQRs). Categorized data are expressed as numbers and percentages. The degree of interobserver agreement was evaluated by Fleiss’ κ values: κ < 0.40, poor agreement; 0.40 < κ < 0.75, fair to good agreement; and 0.75 < κ < 1.00, excellent agreement^32^. All analyses were performed by SPSS 26 (IBM, NY, USA).

### Ethics approval and consent to participate

Informed consent forms were signed by the parents and ethics approval was approved by the institutional review boards of the Children’s Hospital of Fudan Uninversity.

## Conclusion

In conclusion, we described the manifestations on chest CT of CGD patients. CT images of patients with CGD demonstrated a variety of pulmonary abnormalities according to the different infectious pathogens and courses of disease. The main infectious organisms had unique imaging features that could be used to help obtain accurate diagnoses and prevent further pulmonary impairment. CT scans might help in making early and timely diagnoses of lung disease in CGD and could benefit affected patients by aiding in the development of more rigorous treatment.

## Data Availability

The datasets during and/or analysed during the current study available from the corresponding author on reasonable request.
